# Materials, energy, water, and emissions nexus impacts on the future contribution of PV solar technologies to global energy scenarios

**DOI:** 10.1038/s41598-019-55853-w

**Published:** 2019-12-17

**Authors:** Ayman Elshkaki

**Affiliations:** 1 Institute of Geographic Sciences and Natural Resources Research, Chinese Academy of Sciences, 11A Datun Road, Chaoyang District, Beijing, 100101 P.R. China; 2 University of Chinese Academy of Sciences, Beijing, 100049 P.R. China; 3 Key Laboratory of Carrying Capacity Assessment for Resource and Environment, Ministry of Natural Resources, Beijing, 100083 P.R. China

**Keywords:** Energy policy, Energy supply and demand, Sustainability

## Abstract

PV technologies are increasingly making significant contribution to global energy generation (GEG), attributed to their high potential of increasing efficiency, cost reduction, and improving energy security. These technologies however rely on metals that are identified as critical due to risks associated with their supply, and other materials that require energy and water for their production. In this paper, a comprehensive assessment of required materials for PV technologies, an analysis of their materials inflows, outflows, and stocks, an estimate of their maximum contribution to global energy scenarios (GES), and an estimate of energy and water required for their material production and associated CO_2_ emissions under the nexus approach, have been carried out using a dynamic material flow-stock model. A total of 100 energy-material nexus scenarios, which combines 10 GES and 10 materials scenarios, have been analysed. Results indicate that although most GES are difficult to be realized under current PV technologies market share and condition; these technologies could make significant contribution to GEG in future. The three commercial thin-film PV technologies could produce between 3% and 22% of electricity generation in IEA-450 scenario. Energy required for PV materials production is expected to reach between 5.9% and 11.8% of electricity generated (EG) by PV solar and between 0.76% and 1.52% of total EG in IEA-450 scenario by 2050. CO_2_ emissions associated with material production are expected to be between 0.94% and 2.2% of total CO_2_ emissions in IEA-450 scenario by 2050.

## Introduction

Several global energy and climate scenarios have been developed recently^1–10^. These scenarios are driven by environmental sustainability, current and planned policies, energy security, or market mechanisms. The IEA-450 scenario of the International Energy Agency (IEA) is mainly driven by environmental sustainability while the New Policy scenario is driven by current and planned policies^4^. The Energy [R]evolution and Advanced Energy [R]evolution scenarios developed by Greenpeace and other organizations are driven by sustainable energy supply^5^. Symphony and Hard Rock scenarios developed by the World Energy Council (WEC) are driven mainly by environmental sustainability and energy security while Modern Jazz scenario is mainly driven by market forces^6,7^. Fossil fuel firms such as Statoil and Shell also developed scenarios including Statoil Renewal scenario, which focuses on developments consistent with a 50% probability of limiting global warming to 2°^9^, and Shell New Sky scenario, which is the most optimistic scenario in terms of climate outcomes among their scenarios^10^.

Energy demand and supply technologies vary among these scenarios including those aimed at achieving CO_2_ emissions reduction target. PV solar technology is one of the main energy supply technologies in all these scenarios, although with different market share. However, it is argued that all global energy scenarios (GES) have failed to account for PV solar full potential (i.e. the highest level of technology growth)^11,12^, which may affect innovation and development investment and global climate change mitigation efforts^11^. The authors argued that if scenarios such as those developed by the International Energy agency (IEA) recognize the possibility of the fast growth of solar technology, investors and governments will be encouraged to take optimistic view of the potential of PV market. It is also argued that IEA scenarios are conservative, do not analyse technology convergence (i.e. the combined impacts of complementing technologies that could accelerate the transition to renewables), their models for technological advancement are limited, and they do not consider emerging technologies such as perovskite solar cells^11^.

This paper is aimed at analyzing the future contribution of PV solar to several GES, and the maximum potential of PV solar in global electricity generation (GEG), in terms of available technologies, technological advancement, and resources requirements. It is also aimed at analyzing the energy and water required for PV solar materials production and associated CO_2_ emissions. GES analyzed in this study have been developed by international agencies^1–7^, academic institutes^8^, and fossil fuel firms^9,10^, and among those investigated by Carrington and Stephenson^11^. The analysis is motivated by the current discussion on energy-material nexus, which indicates that most global energy scenarios maybe constraints by low carbon technologies requirements for critical materials, and that materials production requires a large amount of energy^13–26^.

GES are produced using energy and integrated assessment models, which either use optimization techniques^8^ or use simulation models^4^ to produce energy mix. Some models use predefined energy mix that is compatible with scenario target. For example, the simulation model used by Greenpeace^5^ assumes growth rates for population, GDP, specific energy demand and deployment of renewable energy technology and it requires a consistent exogenous definition of feasible developments in order to meet the targets. Although these models include materials demand as part of the industrial sector energy demand, none of them include material demand and possible constraints related to their availability and production capacity on the supply side of energy^27–29^. It is argued that energy and integrated assessment models assumptions on inputs, functions, and parameters including technologies cost, which have significant impacts on models outcomes, are normative and reflect modelers’ understanding^30^. It has been pointed out that some models, for example, include a high learning rate of CSP, while others either assume a low learning rate or do not include the technology as an option based on specific narratives^30^. From the materials side, costs of material in some PV technologies module are too low compared to other costs including other components of module cost, balance of system cost, or variable cost^31^ and would not have direct impact on technology choice. Therefore, increasing technologies efficiency and reducing their cost are not enough to determine these technologies contribution to GES.

Material scenario studies have focused on investigating material requirements for PV technologies and potential contribution of these technologies to GEG. Studies addressing material requirements for PV technologies^13,15,22,24,32,33^ are mostly based on dynamic modelling approach and their results vary due to the use of specific energy and technology scenarios, in addition to other assumption on the dynamic factors in their models including technology material content (MC), and life time (LT). Although these scenarios studies provide useful information on future requirements of metals for low carbon technologies, they are limited to specific GES, fixed exogenous market share (MS) of PV solar and its sub-technologies, and discuss complexities arise from metals resource availability and production capacity. Sub-technologies market may change in the next few decades however, and complexities associated with required metals for low carbon technologies are not limited to resources availability or production capacity but also related to the fact that most of these metals are companion metals (i.e. produced with other host metals)^34,35^ and increasing their production is constraints by technology, market price, and demand for host metal, and may result in unintended consequences^36,37^. In addition, most of these metals are produced in limited number of countries and the spread of PV solar and other renewable technologies may shift current geopolitics of fossil fuel to new dependency on critical materials^38^.

Studies addressing potential contribution of PV solar, or PV solar specific technology vary in their approaches and results^16,39–42^. Most of these studies are using static approach, and their analysis either does not include the dynamic mechanisms of metals demand and supply, which would be extremely important especially if the analysis would be carried out on the long term, including the dynamics of sub-technologies MC, metals supply from secondary sources (recycling potential (RP)), and technologies LT, or limited in the number of included technologies. The evaluation of these technologies is based on historical growth rate in all metals production^16^, resources availability^39,40^, production capacity^39,41^, or host metals production^42^. It is indicated recently that there is a high uncertainty in the data and methodologies used to assess future availability of materials required for technologies^43^, in addition to the assumptions on technical characteristics of technologies, and availability of secondary sources.

In this paper, a total of 100 energy-material nexus scenarios have been illustrated. These scenarios are designed to investigate the impacts of different factors (MS, MC, RP, and LT) on metals demand, and (resources availability, production capacity, and coproduction) on metals supply, and ultimately possible realization of GES. The evaluation is carried out based on a comparison between metals cumulative demand (CD) and their global reserves, metals average annual demand (AAD) and their global production, and required production of host metals and their historical production growth rate. Based on this analysis, the maximum contribution of each technology is estimated. In addition, estimates for energy and water required for materials production and associated CO_2_ emissions, and a discussion on perovskite solar cells have been included.

## Methodology

The first part of the methodology describes the 10 GES used in the analysis. These scenarios provide information on PV solar cumulative installed capacity, which is used as a basis of the subsequent calculations. In the second part, the dynamic material flow model, the 100 material-energy nexus scenarios, and the assumptions made for the model parameters have been described. This includes the estimates of PV solar annual installed capacity, PV solar sub-technologies annual installed capacities, and the annual inflows, discarded outflows, and stocks of metals. In addition, it describes the main parameters used in the model including the sub-technologies MS, their MC, and their LT. It also describes the basis of the evaluation of 10 GES in terms of production capacity and resources availability. The third part describes metals coproduction and the basis of the estimates of the host metals production as a result of the companion metals demand, which is also used in the evaluation of GES. The last part of the methodology describes the estimates of energy, water, and CO_2_ emissions associated with metals production.

### Energy scenarios

**Figure 1:**
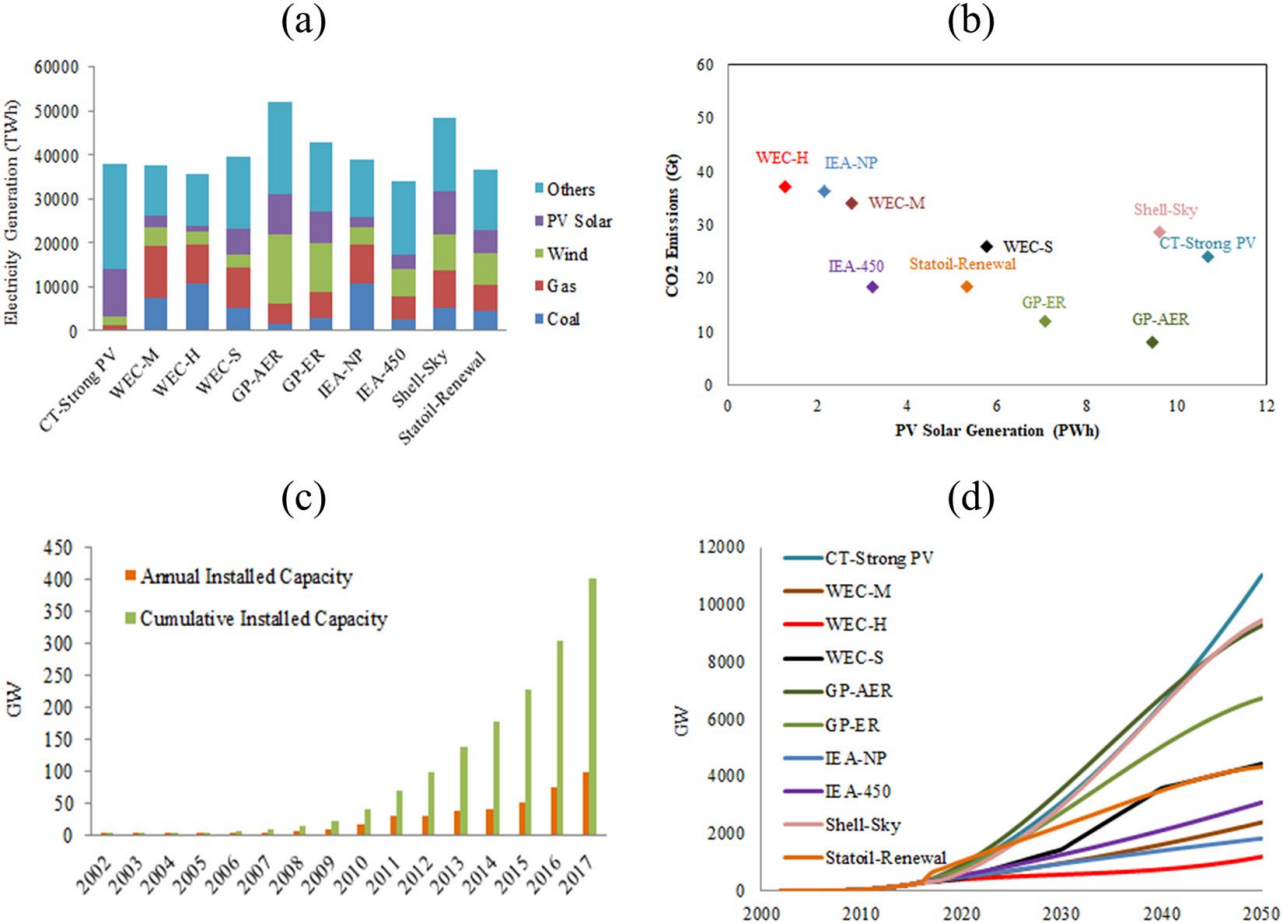
(**a**) Total electricity generation (EG) and electricity mix in the GES; (**b**) PV solar EG and CO_2_ emissions associated with each scenario; (**c**) historical PV annual and cumulative installed capacity and (**d**) future PV installed capacity in GES.

Several GES (Table 1) have been developed, which provide several estimates for future demand and supply of electricity, and consequently associated CO_2_ emissions. These scenarios have been chosen for the analysis mainly because they cover wide range of estimates of PV solar technologies contribution to energy scenarios, driven by different factors, representative of different modelling techniques, and representing the different views of the international organizations, academic institutes, and fossil-fuel firms. Figure 1a shows total electricity generated by each technology in 10 GES in 2040 and Fig. 1b shows CO_2_ emissions associated with these scenarios. Figure 1c shows annual and cumulative installed capacity of PV technologies in last 15 years, and Fig. 1d shows future cumulative installed capacity in GES up to 2050. Data on EG, EG by PV technologies (EG-PV), and CO_2_ emissions associated with GES are obtained from several reports published by scenarios developers^4–10^. Some of these sources provide data up to 2050 and others up to 2040. EG-PV and installed capacity (IC-PV) data given for each scenario are either each 5 or 10 years. Missing data up to 2050, and annual EG-PV and IC-PV are linearly estimated, as the growth of both in the original scenarios is linear and to be in line with assumptions made in other studies^15^. The analysis is carried out from 2015 to 2050. The target year is set to 2050 mainly due to the long PV solar technologies LT and to allow for analysing the impacts of factors that may not appear on shorter simulation time including recycling. Highest electricity demand is expected in GP-AER scenario, while the lowest is expected in IEA-450 scenario. Highest EG-PV and highest market share of PV solar technology are expected in CT-Strong PV scenario and the lowest are expected in WEC-H scenario. Highest CO_2_ emissions are expected in WEC-H scenario and the lowest are expected in GP-AER scenario.

**Table 1:** Global Energy Scenarios used in the analysis.

Global energy Scenarios	Short name
Carbon Tracker 2017 Strong PV scenario	CT-Strong PV
Greenpeace Energy Outlook 2015 Advance Energy [R]evolution scenario	GP-AER
Greenpeace Energy Outlook 2015 Energy [R]evolution scenario	GP-ER
IEA World energy Outlook 2016 450 scenario	IEA-450
IEA World energy Outlook 2016 New Policy scenario	IEA-NP
World Energy Council Scenarios 2016 Hard Rock scenario	WEC-H
World Energy Council Scenarios 2016 Modern Jazz scenario	WEC-M
World Energy Council Scenarios 2013 Symphony scenario	WEC-S
Shell 2018 New Sky scenario	Shell-Sky
Statoil Energy Perspective 2017 Renewal scenario	Statoil-Renewal

### Dynamic material flow analysis

**Figure 2:**
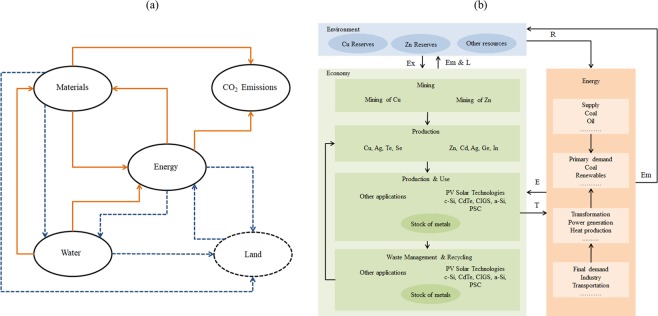
(**a**) Material, energy, water, land, and CO2 emissions nexus; the solid lines and circles are included in the analysis, and dotted lines and circles are excluded, (**b**) MFA system (metals main processes, flows, and stocks in the economy and environmental systems), and the link to the energy supply, demand, and transformation. Ex refers to extraction, Em & L refers to emissions and losses, E refers to energy, T refers to technology, Em refers to emissions, and R refers to resources.

Figure 2a illustrates material, energy, water, land, and CO_2_ emissions nexus. Energy is required for water extraction, distribution, and treatment, while water is required for power generation, biofuel production and other energy processes^4^. Materials are required for energy production technologies^13–22^ and water production, distribution, and treatment, while energy and water are required for all material production processes^26^. Material-energy-water nexus is also linked to land and climate change. In the analysis described in this paper, the solid lines and circles in Fig. 2a (material for energy, energy and water for materials, water for energy, and associated CO_2_ emissions) have been included in the analysis, and dotted lines and circles have been excluded. Figure 2b illustrates MFA system (economy and environment subsystems) and the concept of energy-material nexus related to PV solar. It shows the main processes (mining, production, use, and waste management), flows (extraction, emissions, losses, mined metals, products, and waste), and stocks of metals in the economic and environmental systems (resources, stock-in-use, stock in landfill sites). It also shows the energy supply, demand, and transformation, as well as the extracted resources and emitted flows in the energy system. The main flow from the energy system is the energy required for all processes in materials life cycle and the main flow from the materials system is the technologies required for energy generation.

Metals annual and cumulative demands are estimated based on a dynamic material flow-stock model^44,45^. Metals inflow (annual demand) with each PV sub-technology is estimated as given by Eq. (1) and metals cumulative demand between 2015 and 2050 as given by Eq. (2).

1 $${F}_{M,ST}^{in}(t)=\,{F}_{T}^{in}(t)\times \delta (t)\times M{C}_{M}(t)$$

2 $$C{D}_{M,ST}(t)=\,\mathop{\sum }\limits_{i=1}^{n}{F}_{M,ST}^{in}(t)$$

*T*, *ST*, and *M* in equations refer to technology, sub-technology, and metals.

Two scenarios are used for Material content (*MC*_*M*_ in Eq. (1)). The first assumes constant materials contents (MC constant scenario) based on data given in^19,22^, which are compiled based on different values reported in several studies, and between maximum and minimum values reported in literature (Fig. S1). The second assumes changing MC (MC changing scenario) and is based on assumptions for technical aspects of technologies (Eq. (3))^19,32,42,46–50^. PV solar sub-technologies MS is determined by technical, economic, and policy factors, and expected to change overtime, although the magnitude and timing of the change is uncertain^19^. Therefore, two scenarios are used for PV sub-technologies MS (*δ* in Eq. (1)). The first (MS1 scenario) assumes a decrease in Si based technologies MS with a minimum of 50% and the three TF technologies (amorphous silicon (a-Si), cadmium telluride (CdTe), and copper indium gallium diselenide (CIGS)) have equal shares. This is in line with assumptions made in other studies, which assumes a decrease in the Si based technologies MS and an increase of thin film technologies MS, with an equal MS of different thin film technologies^15,17,19,22^. The second (MS2 scenario) is based on a scenario guided by information given in^51^. The two MC and MS scenarios are listed in Table 2 and assumptions in other studies are listed in Tables S1 and S2 in SI.

3 $${M}_{M}(t)=\,\frac{L\times \rho \times W}{\mu \times STC\times PR\times U}(1-R)$$

Where *L* is absorber layer thickness, *ρ* is layer density, *W* is metal mass fraction in layer, *U* is metal utilization rate, *R* is metal recovery rate, *µ* is conversion efficiency, *STC* is irradiation, and *PR* is performance ratio.

**Table 2:** Metals contents (kg/MW) and market shares of PV solar technologies (%).

PV solar technologies	c-Si	CdTe		CIGS			a-Si	Others
	Ag	Cd	Te	In	Ga	Se	Ge	
Constant MC	80	85	97.5	23	7.5	45	73	
Changing MC - 2030	10	31	35	11	3	23	35	
Changing MC - 2050	1	12	14	6	1	11	14	
Market Share (MS1) - 2030	50	16.67		16.67			16.67	0
Market Share (MS1) - 2050	50	16.67		16.67			16.67	0
Market Share (MS2) - 2030	79.8	4.9		6.8				8.5
Market Share (MS2) - 2050	62.9	4.4		12				20.7

Annual installed capacity for PV technology (inflow) is estimated based on cumulative installed capacity (*S*_*T*_) given in GES scenarios and annual discarded flow of old annual installed capacity (*F*_*T,D*_^*out*^) (Eq. (4)), which is estimated based on the inflow and technologies LT (Eq. (5)).

4 $${F}_{T}^{in}(t)={S}_{T}(t)-{S}_{T}(t-1)+{F}_{T,D}^{out}(t)$$

5 $${{F}^{out}}_{T,D}(t)={F}_{T}^{in}(t-LT)$$

PV solar technologies LT is a significant factor in determining the discarded outflow of PV solar technologies and consequently the supply of materials from secondary and primary sources. Several values of the technologies life time have been reported in literature, which range from 20 to 30 years^15,17,23,24,52,53^. Although it’s preferable to use lifetime distribution instead of fixed lifetime for products with short lifetime and high variations^15^, PV solar technologies have long lifetime and low variations. Therefore a fixed life time has been used in the analysis. To address the uncertainty associated with the lifetime and to examine the impacts of both shorter and longer LT, combined with the impacts of other factors such as MC, two values for the LT have been used. The lifetime is assumed to 20 years (LT1) in some scenarios, and 30 years (LT2) in others.

Metals primary inflow with each sub-technology is estimated based on metals inflow (annual demand) with each sub-technology and discarded metals outflow multiplied by their recycling rate (RR) (Eq. (6)).

6 $${F}_{M,ST,prim}^{in}\,(t)={F}_{M,ST}^{in}(t)-({{F}^{out}}_{M,ST,D}(t)\times RR)$$

Although recycling rates are low for most critical metals used in PV technologies^20,54^, two scenarios for secondary supply (recycling potential (RP)) are used in the analysis. The first assumes no recycling for metals used in the PV technologies (WOR scenario) and the second assumes recycling rate of 50% for all metals (WR scenario). Discard metals outflow is estimated as delayed inflow (Eq. (7)), and metals stocks are estimated based on metals inflows and outflows (Eq. (8)). If metal emissions occur during use, the flow is estimated as fraction (α) of stock and the discarded outflow should be corrected (Eqs (9) and (10))^44^.

7 $${{F}^{out}}_{M,ST,D}(t)={F}_{M,ST}^{in}(t-LT)$$

8 $$\,{S}_{M}(t)={S}_{M}(t-1)+{F}_{M}^{in}(t)-{F}_{M}^{out}(t)$$

9 $${F}_{M\,E}^{out}(t)=\alpha \times {S}_{M}(t)$$

10 $${F}_{M,ST,D}^{out}(t)={F}_{M,ST}^{in}(t-LT)-\mathop{\sum }\limits_{i=1}^{{L}_{U}}\alpha \times {F}_{M,ST}^{in}\,(t-LT)\times {(1-\alpha )}^{i-1}$$

The evaluation of GES in terms of metals resources availability and production capacity, and host metals production capacity is based on a comparison between cumulative demand (CD) and reserves, average annual demand (AAD) and current production, and average required production of host metals and their historical average production growth rate. The average annual demand has been estimated as an average value of the annual demand between 2015 and 2050 (36 years). The average annual demand, which would cover the entire time, is used instead of annual demand due to the possibility of having low annual demand at the beginning of the simulation time while having very high annual demand by the end of the simulation time that would lead to very high values compared to current production and host metals production. Reserves and production estimates for PV technologies metals and their host metals are obtained from different sources^55–59^. A sensitivity analysis for indium and tellurium has been carried out to examine the impact of resources availability and production capacity on the maximum contribution of TF technologies. The estimates used in this analysis and other studies are indium resources reported by USGS, which has stopped reporting these resources in recent years. Mudd *et al*.^60^ have reported estimates of indium reserves of 33100 Mg, about three times USGS estimates, and an annual growth rate of 4.7% for Te production.

The combined scenarios used in the analysis are listed in Table 3. Scenario 1 to 6 examine the impacts of MC, RP, and LT, scenarios 7 and 8 examine the impact of uncertainty in resources and production capacity, and scenarios 9 and 10 examine the impacts of technologies MS.

**Table 3:** Combined scenarios used in the analysis.

Scenario No.	Combined scenarios
*SC 1*	*10 GES, Market Share 1, MC Constant, Life Time 1 (20 years), Without Recycling*
*SC 2*	*10 GES, Market Share 1, MC Changing, Life Time 1 (20 years), Without Recycling*
*SC 3*	*10 GES, Market Share 1, MC Constant, Life Time 1 (20 years), With Recycling*
*SC 4*	*10 GES, Market Share 1, MC Changing, Life Time 1 (20 years), With Recycling*
*SC 5*	*10 GES, Market Share 1, MC Changing, Life Time 2 (30 years), Without Recycling*
*SC 6*	*10 GES, Market Share 1, MC Changing, Life Time 2 (30 years), With Recycling*
*SC 7*	*10 GES, Market Share 1, MC Constant, Life Time 1 (20 years), Without Recycling, S*
*SC 8*	*10 GES, Market Share 1, MC Changing, Life Time 2 (30 years), With Recycling, S*
*SC 9*	*10 GES, Market Share 2, MC Constant, Life Time 1 (20 years), Without Recycling*
*SC 10*	*10 GES, Market Share 2, MC Changing, Life Time 1 (20 years), Without Recycling*

### Metals coproduction

Most PV technologies metals are companion metals, which are produced in small quantities with other host metal. About 95% of In, 70% of Ge, 100% of Cd, and 36% of Ag are produced from Zn deposits^60–64^, and about 22% of Ag and more than 90% of Te and Se are produced from Cu deposits^64,65^. Although these metals are mainly produced with zinc and copper, In could be found with tin and tungsten but it is usually difficult to extract it economically^66^ and Te could be extracted on its own but in small quantities^67^. Based on the 2016 production data for Zn, In, Ge, Cd, Ag, Cu, Te, and Se^57^, about 51.3 g of In, 7.0 g of Ge, 1897 g of Cd, and 734 g of Ag are produced with each ton of Zn, and about 20.4 g of Te, 162 g of Se, and 281 g of Ag are produced with each ton of Cu (Table 4). These values have been used in the analysis. However, historically the ratios of companion metals to host metals production have changed (Fig. S2). Although theoretically Te production for each ton of Cu, and Ge production for each ton Zn could be increased due to the difference between actual production ratios and maximum theoretical ratios (based on their concentration in deposits), historically production ratios of the two metals to their hosts have decreased. The situation is different for In as historically it was possible to increase production ratio (Fig. S2 and other literature^37,60^). Maximum theoretical ratios of In, Ge and Cd to Zn, and Te and Se to Cu based on their concentrations in deposits given in^68,69^ and their recovery rates are listed in Table 4. Historical world production of Cu, Zn and their companion metals appears in SI (Fig. S3).

**Table 4:** Metals concentration in deposits, maximum theoretical ratios of companion to host metals, actual production ratios of companion to host metals, and recovery rate.

	Zn	Cd	In	Ge	Ag
Concentration in deposit (%)	4	0.02	0.002	0.002	
Maximum theoretical production of CM to Zn ratio (g CM/ton Zn)		5000	100	500	
Production in 2016 (Mg)	12.6 M	23900	680	126	25700
Global ratio of CM to HM production (g CM/ton Zn)		1897	51.3	7.0	734
Recovery (%)		38	51	1.4	
	**Cu**	**Te**	**Se**		**Ag**
Concentration in deposit (%)	0.8	0.0002	0.0005		
Maximum theoretical production of CM to Cu ratio (g CM/ton Cu)		250	670		
Production in 2016 (Mg)	20.1 M	410	3270		25700
Global ratio of CM to HM production (g CM/ton Cu)		20.4	162		281
Recovery (%)		8.2	24.2		

### Energy and water required for materials production and associated CO_2_ emissions

Energy and water required for the production of PV technologies materials and associated CO_2_ emissions are estimated based on annual amount of materials required for these technologies, obtained using the dynamic material flow-stock model, and their energy, water, and CO_2_ intensities (Eqs (11–13)). In addition to the materials listed in Table 2, required amounts of Cu, Ni, Cr, Pb, Mo, Sn, Zn, Al, Fe, and concrete are included in these estimates. The amount of energy required for metals production is mainly determined by metals ore grade, metals production routes, the efficiency of energy use in metals extraction and production, and the demand for the metals. The most relevant materials, among those used in PV solar technologies, in terms of energy, water, and CO_2_ emissions are Al, Fe, and concrete, not because their energy intensities are expected to increase (as a result of decreasing ore grade or lower energy efficiency) but mainly due to the quantities used in these technologies. Several values for Al, steel, and concrete intensities of PV solar have been reported^20,70,71^. Minimum, maximum, and average Al intensity in PV is assumed 19 Mg/MW, 33 Mg/MW, and 26 Mg/MW, steel intensity is assumed 178 Mg/MW, 318 Mg/MW, and 251 Mg/MW, and concrete intensity is assumed 672 Mg/MW, 2846 Mg/MW, and 1823 Mg/MW based on values reported in^71^ for average literature data. Energy intensities for metals reported in^72^ and for concrete reported in^52^ have been used. Although ore grades for most metals are expected to decrease overtime, the ore grade of Al and Fe is not expected to change^73^. Consequently the main determinant factor for the energy required for Al and steel production is the efficiency of energy use which is expected to improve overtime^73^. It is known that theoretical energy is several times less than actual energy used in metals production^73^. Energy intensities for Al, steel, and concrete are assumed to be reduced annually by 0.66%, 0.93%, and 1.05% respectively based on a scenario given in^52^. Based on these scenarios, energy intensities are expected to be reduced by 21%, 27%, and 30% for the three materials respectively. Compared to the theoretical limits^73^, energy efficiency for Al and steel could be reduced more than what is assumed in the scenario. Water intensities for metals reported in^74^ and for concrete reported in^75^ have been used. In addition to required water for metals production, water required for PV technologies operation is estimated based on the electricity production by PV solar reported in GES and water requirement reported in^76^ for consumption and withdrawal (Eq. (12)). CO_2_ emission factors for metals production reported in^72^ and for concrete reported in^77^ have been used, which is slightly lower than a value given in^70^. Ultimately this value will be determined by the geographical location of PV solar installation and the technologies and energy mix used for the production of concrete. For Al, steel, and concrete, CO_2_ emission factors are assumed to be reduced annually, based on a scenario similar to the one used for energy reduction. Based on the scenario, CO_2_ emissions intensities are expected to be reduced by 1600 kg CO_2_/ Mg of Al, 400 kg CO_2_/Mg of steel, and 61 kg CO_2_/Mg of concrete. Compared to the expected reduction reported in^73^, CO_2_ emissions of Al and steel production could be reduced more than what is assumed in the scenario. The energy, water, and CO_2_ emissions analysis is carried out for IEA-450 scenario and ***SC1*** and ***SC2***.

11 $$E(t)=\,\mathop{\sum }\limits_{i=1}^{n}{F}_{Mi,ST}^{in}(t)\times {E}_{Mi}(t)$$

12 $$W(t)=\,\mathop{\sum }\limits_{i=1}^{n}{F}_{Mi,ST}^{in}(t)\times {W}_{Mi}(t)+E(PV)(t)\times W(t)$$

13 $$Em(t)=\mathop{\sum }\limits_{i=1}^{n}{F}_{Mi,ST}^{in}(t)\times E{m}_{Mi}(t)$$

## Results

The results include first an analysis of one of the GES; CT-Strong PV, followed by an analysis of the annual demand for metals in all GES, cumulative demand for metals in all GES, coproduction of metals in all GES, and metals inflows, outflows, and stocks, the estimates of PV technologies maximum contribution to future global energy system, and finally the energy and water required for metals production and associated CO_2_ emissions.

### Analysis of CT-Strong PV scenario

**Figure 3:**
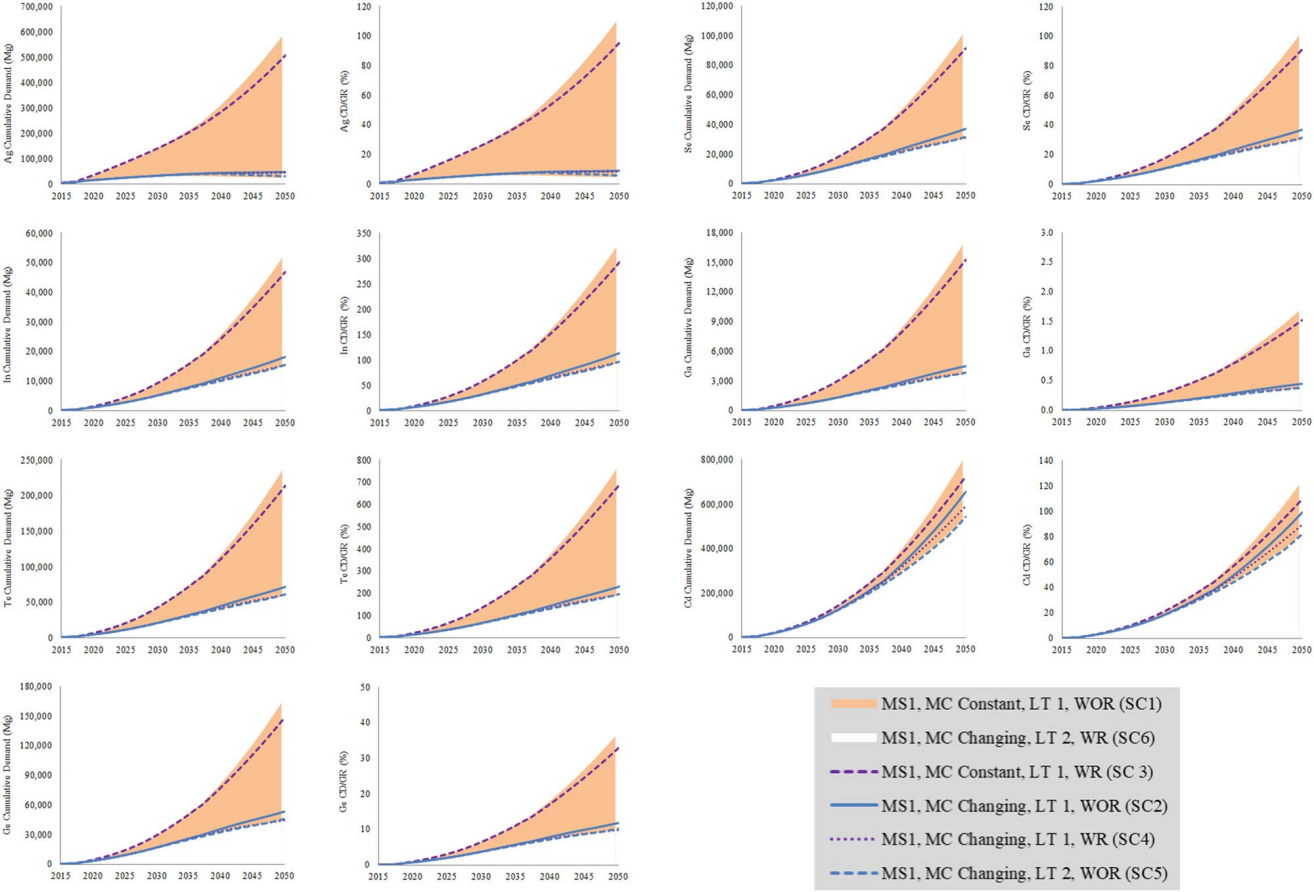
Cumulative demand for metals and cumulative demand compared to global reserves in the CT-Strong PV scenario and six material scenarios.

Highest annual demand for all metals required in PV technologies in 2050 in the 10 GES and two MC scenarios is expected in CT-Strong PV scenario, followed by GP-AER, and Shell-Sky. Similarly, highest cumulative demand between 2015 and 2050 for all metals is expected in CT-Strong PV scenario followed by GP-AER, and Shell-Sky, except for Ag, which has highest cumulative demand in MC changing scenario in GP-AER scenario. Figure 3 shows the results of SC1 to SC6 for CT-Strong PV scenario. The most important factor in determining the metals requirement for PV technologies is MC. Advancement in technology is expected to reduce the risk associated with Ag, Se, and Cd in terms of resources availability, and with In and Ga in terms of production capacity. Although technology LT and metals RR are important, both have similar effect on metals requirements and come after MC. The impact of recycling is similar to the impact of the long LT for all metals except for Ag and Cd. For Ag, the impact of recycling is more than the impact of the long LT, while the impact of recycling is less than the impact of the long LT for Cd.

### Annual demand for metals in all GES

**Figure 4:**
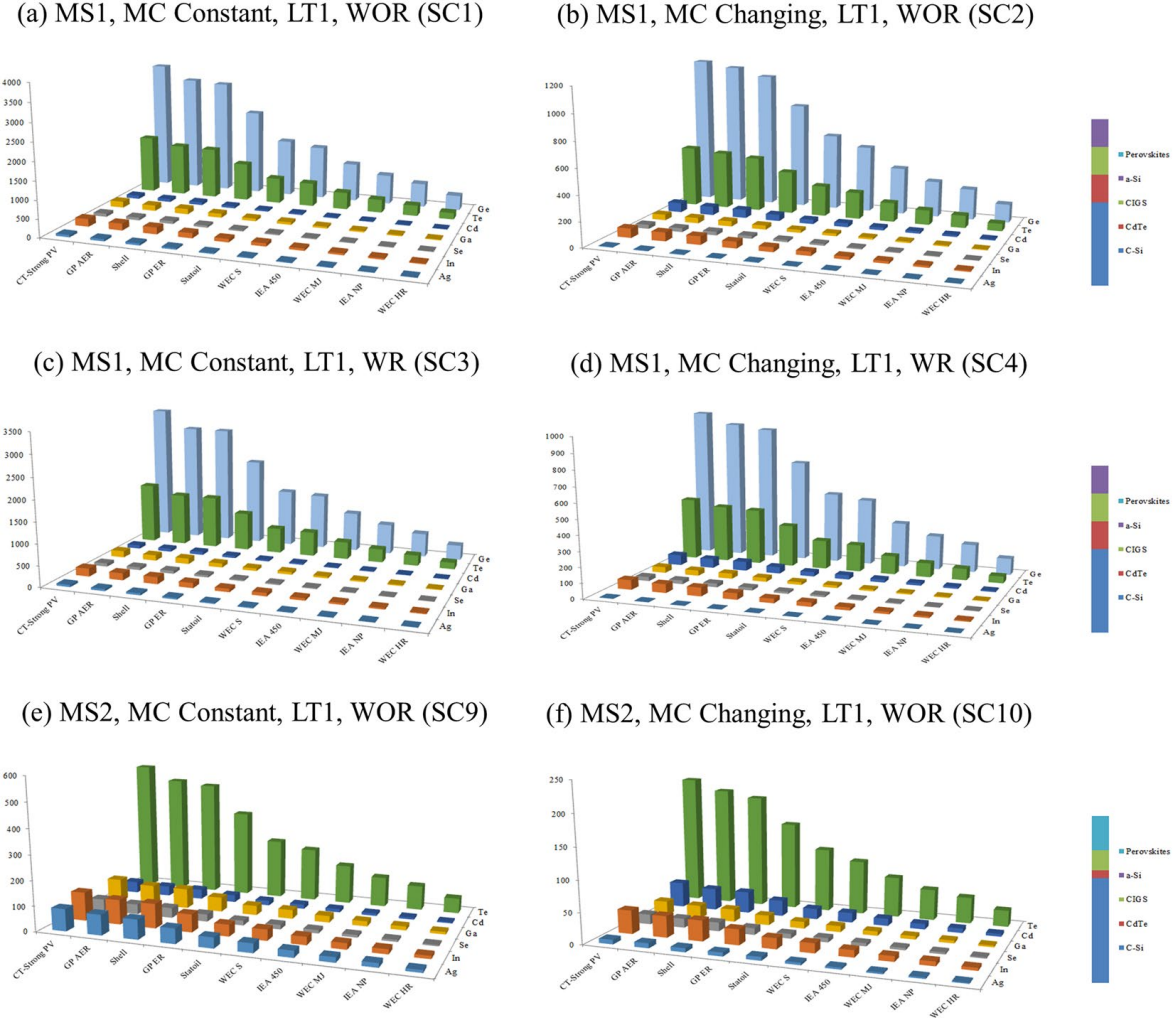
Average annual demands for metals in PV solar technologies compared to current production in GES and different materials scenarios.

In ***SC1***, AAD for Ag, Se, and Cd are not expected to exceed global production in all GES, AAD for In and Ga are expected to exceed global production in CT-Strong PV, GP-AER, Shell-Sky, and GP-ER scenarios, and AAD for Te and Ge are expected to exceed global production in all scenarios (Fig. 4a). Therefore, technologies that require Te and Ge may limit the realization of these scenarios. In terms of production capacity, if MC remains constant, all scenarios are not expected to be realized if CdTe and a-Si are part of the PV market. If CdTe and a-Si can be replaced by other technologies, all scenarios are expected to be realized except CT-Strong PV, GP-AER, Shell-Sky, and GP-ER scenarios. In ***SC2***, AAD for Ag, In, Se, Ga and Cd are not expected to exceed global production in all GES, and AAD for Te and Ge are expected to exceed global production in all GES (Fig. 4b). Even with advances in MC, all GES are not expected to be realized if CdTe and a-Si are part of the PV market as a result of Te and Ge demand. If CdTe and a-Si can be replaced, all GES are expected to be realized.

In ***SC3***, AAD for Ag, Se, and Cd are not expected to exceed global production in all GES, AAD for In and Ga are expected to either exceed or be similar to global production in CT-Strong PV, GP-AER, Shell-Sky, and GP-ER scenarios, and AAD for Te and Ge are expected to exceed global production in all GES (Fig. 4c). Recycling does not change the risk associated with production capacity when MC is constant. In ***SC4***, AAD for Ag, In, Se, Ga and Cd are not expected to exceed global production in all GES, AAD for Ge is expected to exceed global production in all GES, and AAD for Te is expected to exceed global production in all GES except WEC-H, WEC-M, and IEA-NP (Fig. 4d). Recycling even with advances in MC is not expected to change the outcome if a-Si is part of the PV market. Most scenarios are not expected to be realized if CdTe is part of PV market.

In ***SC5***, AAD of Ge, Te, and In, in CT-Strong PV, are expected to be 1012%, 412%, and 63% of their current production, compared to 1176%, 483%, and 74% in the shorter LT. AAD of Ge, Te, and In, in Statoil-Renewal, are expected to be 494%, 198%, 30% of their current production, compared to 610%, 245%, and 37 in the shorter LT. AAD of Ge, Te, and In, in WEC-H, are expected to be 116%, 48.6%, 7.6% of their current production, compared to 146%, 60%, and 9.2% in the shorter LT. In ***SC6***, AAD of Ge, Te, and In, in the CT-Strong PV, are expected to be 966%, 393%, and 60% of their current production compared to 980%, 409%, and 63% in the shorter LT. The results of CT-Strong PV in ***SC5*** and ***SC6*** show that when a recycling system is not available the longer lifetime is better indicated by the relatively high difference in the AAD compared to current production in ***SC5*** for the three metals. When recycling system is available, the difference between the longer LT and shorter LT, indicated by AAD compared to current production in ***SC6***, is relatively low.

In ***SC9***, AAD for Ag, Se, Ga, and Cd are not expected to exceed global production in all GES, AAD for In is expected to exceed global production in CT-Strong PV scenario, and AAD for Te is expected to exceed global production in all GES except IEA-NP and WEC-H scenarios (Fig. 4e). If MC remains constant, all scenarios are not expected to be realized, if CdTe is part of PV market, except IEA-NP and WEC-H scenarios. If CdTe can be replaced, all scenarios are expected to be realized except CT-Strong PV scenario. In ***SC10***, AAD for Ag, In, Se, Ga and Cd are not expected to exceed global production in all GES, AAD for Te is expected to exceed global production in CT-Strong PV, GP-AER, Shell-Sky, Statoil-Renewal, and GP-ER scenarios (Fig. 4f). Even with advances in MC, most scenarios are not expected to be realized if CdTe is part of the PV market. If CdTe can be replaced, all scenarios are expected to be realized.

### Cumulative demand for metals in all GES

**Figure 5:**
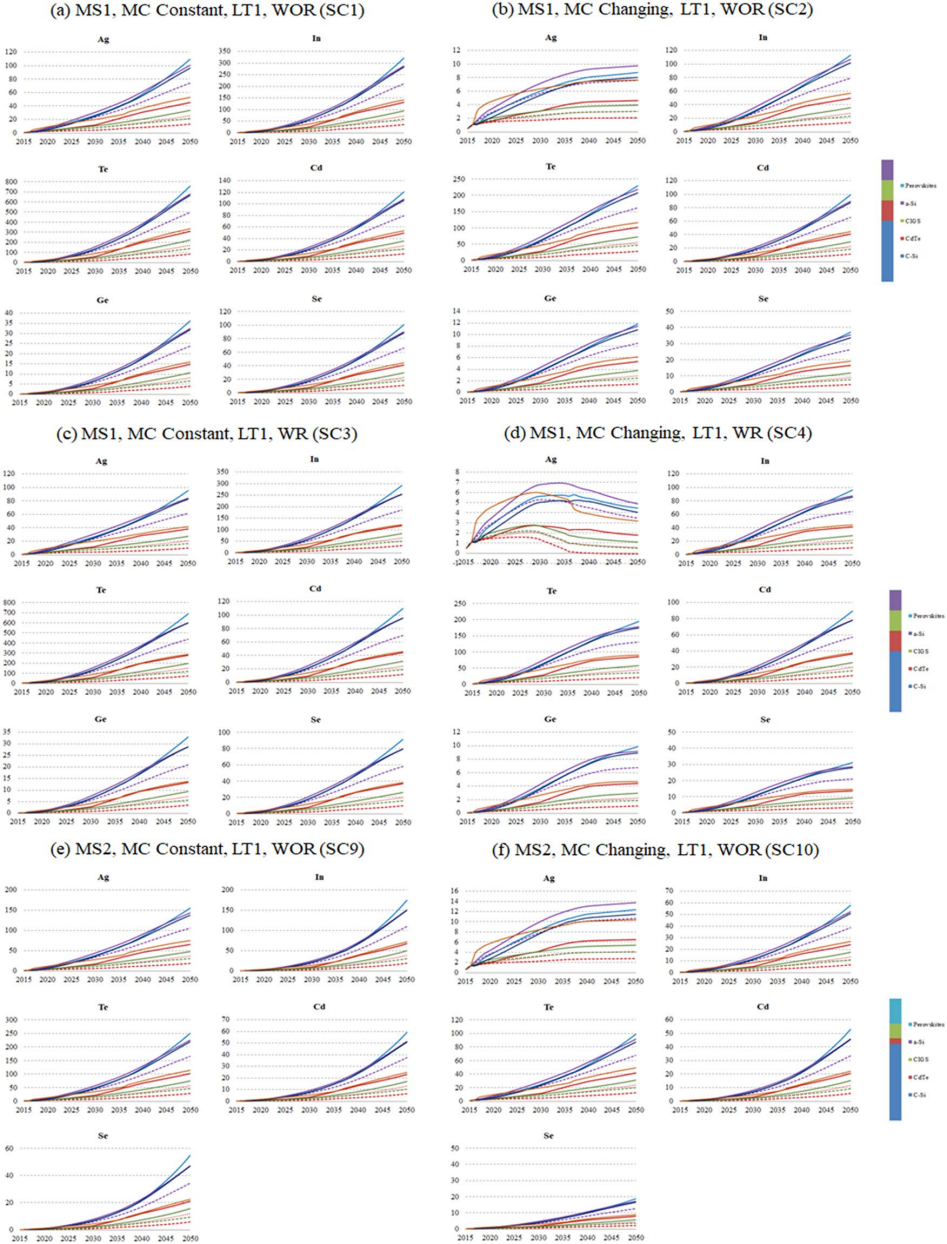
Cumulative demands for metals compared to global reserves in GES and different materials scenarios.

In ***SC1***, CD for Ga and Ge is not expected to exceed global reserves in all GES, CD for Se is expected to exceed global reserves in the CT-Strong PV scenario only, CD for Ag is expected to exceed global reserves in CT-Strong PV and GP-AER scenarios, CD for Cd is expected to exceed global reserves in CT-Strong PV, GP-AER, and Shell-Sky scenarios, CD for In is expected to exceed global reserves in CT-Strong PV, GP-AER, Shell-Sky, Statoil-Renewal, GP-ER, and WEC-S scenarios, and CD for Te is expected to exceed global reserves in all scenarios except WEC-H scenario (Fig. 5a). In terms of resource availability, if MC remains constant, most of the scenarios are not expected to be realized. The only scenario that can be realized is WEC-H scenario if CdTe is part of the PV market. If CdTe can be replaced, several scenarios are expected to be realized including WEC-M, WEC-H, IEA-NP, and IEA-450 scenarios. In ***SC2***, CD for Ga, Ge, Se, Ag, and Cd is not expected to exceed global reserves in all GES, CD for In is expected to exceed global reserves in CT-Strong PV, GP-AER, and Shell-Sky scenarios, and CD for Te is expected to exceed global reserves in all scenarios except WEC-M, WEC-H, IEA-NP, and IEA-450 scenarios (Fig. 5b). With advances in MC, the scenarios that are expected to be realized, if CdTe is part of the PV market, are WEC-M, WEC-H, IEA-NP, and IEA-450 scenarios. If CdTe can be replaced, all scenarios are expected to be realized except CT-Strong PV, GP-AER, and Shell-Sky scenarios.

In ***SC3***, CD for Ag, Ga, Ge, and Se are not expected to exceed global reserves in all GES, CD for Cd is expected to exceed global reserves in the CT-Strong PV scenario only, CD for In is expected to exceed global reserves in the CT-Strong PV, GP-AER, Shell-Sky, Statoil-Renewal, GP-ER, and WEC-S scenarios, and CD for Te is expected to exceed global reserves in all GES except WEC-H scenario (Fig. 5c). Recycling is expected to make some differences for Ag, Se, and Cd, however it is not expected to make differences for In and Te. In ***SC4***, CD for Ga, Ge, Se, Ag, In and Cd are not expected to exceed global reserves in all GES, and CD for Te is expected to exceed global reserves in CT-Strong PV, GP-AER, Shell-Sky, and GP-ER scenarios only (Fig. 5d). Recycling is expected to make a difference for In and small difference for Te. In ***SC5***, higher LT of PV technologies has reduced CD for Ag, In, Se, Ga, Ge, Te, and Cd by 5.7%, 14.9%, 14.5%, 14.7%, 13.9%, 14.7%, and 17.2% in CT-Strong PV respectively. Although the change in LT is expected to reduce CD for all metals, CD for Te still exceeds global reserves and CD for In is 96% of global reserves. In Statoil-Renewal, higher LT has reduced CD for the same metals by 6.3%, 18.7%, 18.8%, 19.4%, 18.9%, 19.3%, 21.4% respectively. In WEC-H, higher LT has reduced CD for the same metals by 10.2%, 17.5%, 18.5%, 18.7%, 20.4%, 18.5%, 14.2% respectively. This indicates that when a recycling system is not available, the longer LT is better as CD for all metals and in the three scenarios is expected to be reduced by between 5.7% and 21.4%. In ***SC6***, higher LT has increased CD for Ag by 25%, and reduced CD for In, Se, Ga, Ge, Te, and Cd by only 4.5%, 3.3%, 3.6%, 1.4%, 3.9%, and 10.3% in CT-Strong PV respectively. Although the change in LT is expected to reduce CD for most metals, CD for Te still exceeds global reserves and CD for In is 92% of global reserves. This indicates that when MC is decreasing at high rate and a recycling system is available, shorter LT is better as illustrated by Ag, and the difference between the two LT scenarios is smaller compared to the one when recycling system does not exist as illustrated by the reduction in the CD for all other metals.

In ***SC9***, CD for Cd and Se are not expected to exceed global reserves in all GES, CD for Ag and In are expected to exceed global reserves in CT-Strong PV, GP-AER, Shell-Sky, and GP-ER scenarios, and CD for Te is expected to exceed global reserves in CT-Strong PV, GP-AER, Shell-Sky, Statoil-Renewal, GP-ER, and WEC-S scenarios (Fig. 5e). If MC remains constant, most of the scenarios are not expected to be realized. The only scenarios that can be realized, if CdTe is part of the PV market, are WEC-M, WEC-H, IEA-NP, and IEA-450 scenarios. If CdTe can be replaced, several scenarios are expected to be realized including WEC-M, WEC-H, WEC-S, IEA-NP, IEA-450, and Statoil-Renewal scenarios. In ***SC10***, CD for all metals are not expected to exceed global reserves in all GES, however CD for Te is expected to be 100% global reserves in CT-Strong PV, and 90% in the GP-AER and Shell-Sky, and CD for In is expected to be between 50% and 60% of the global reserves in CT-Strong PV, GP-AER and Shell-Sky (Fig. 5f). This indicates that with advances in MC, all GES can be realized, assuming no other demand for Te and In by other use applications.

### Coproduction of metals in all GES

**Figure 6:**
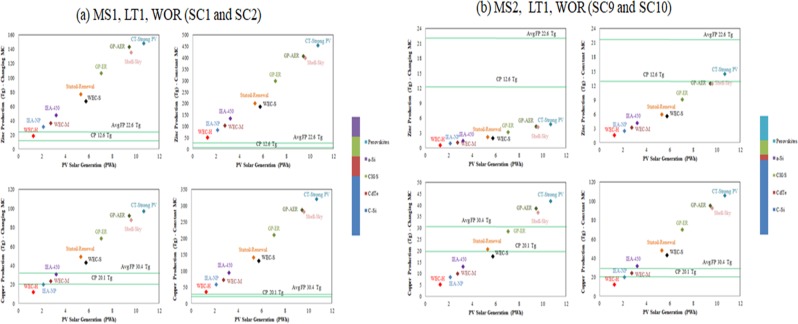
Production of Zn and Cu in *SC1, SC2, S9*, and ***SC10*** for all GES.

In ***SC1***, Zn production as a result of Ge demand in a-Si is expected to be between 50.5 Tg and 454 Tg (between 4 and 36 times current total production of Zn) in all EGS, while it is expected to be between 18 Tg and 148 Tg (between 1.5 and 12 times current total production of Zn) in all EGS in ***SC2*** (Fig. 6a). In ***SC1***, Cu production as a result of Te demand in CdTe is expected to be between 35.6 Tg and 320 Tg (between 1.8 and 16 times the current total production of Cu) in all EGS, while it is expected to be between 12 Tg and 97 Tg (between 0.6 and 4.8 times current total production of Cu) in all EGS in ***SC2*** (Fig. 6a). Based on its historical production, future average Zn production between 2015 and 2050 is expected to be 22.6 Tg, about 1.66 times the current production, while future average Cu production is expected to be 30.4, about 1.6 times the current production. This indicates that in ***SC1***, Zn and Cu production as a result of Ge and Te demand are expected to exceed the average production of Zn and Cu in all EGS. In ***SC2***, Zn production as a result of Ge demand is expected to exceed average Zn production in all EGS except WEC-H, while Cu production as a result of Te demand is expected to exceed the average Cu production in all EGS except WEC-H, WEC-M, IEA-NP, and IEA-450. In this PV market scenario (MS1), Ge and Te demands are expected to require an additional extraction of Zn and Cu in all EGS and MC scenarios, with exception of some GES and MC changing scenario. When the demands for Zn and Cu do not grow at the same level as the demands for the metals required for PV technologies, the extraction of Zn and Cu would be only because of the demands for the companion metals and thus all the environmental impacts associated with that extraction should be allocated for the companion metals required for PV technologies. In ***SC9***, Zn production as a result of In demand in CIGS is expected to be between 1.6 and 14 Tg (between 0.13 and 1.1 times the current total production of Zn) in all GES, while it is expected to be between 0.6 Tg and 4.8 Tg (between 0.05 and 0.4 times current total production of Zn) in all GES in ***SC10*** (Fig. 6b). Cu production as a result of Te demand in CdTe is expected to be between 12.2 Tg and 106 Tg (between 0.6 and 5.3 times the current total production of Cu) in all GES and ***SC9***, while it is expected to be between 5.3 Tg and 41.8 Tg (between 0.3 and 2.1 times current total production of Cu) in all GES and ***SC10*** (Fig. 6b). In the two MC scenarios, Zn production as a result of In demand is not expected to exceed average Zn production in all GES. In the MC constant scenario, Cu production as a result of Te demand is expected to exceed the average Cu production in all GES except WEC-H, WEC-M, IEA-NP, while in the MC changing scenario, Cu production is expected to exceed the average Cu production in CT-Strong PV, GP-AER, Shell-Sky scenarios only. In this market scenario (MS2), the demand for In is not expected to require an additional Zn production in all GES and for the two MC scenarios, while the demand for Te is expected to require additional Cu production in some GES and in the two MC scenarios.

### Analysis of metals inflows, outflows, and stocks

**Figure 7:**
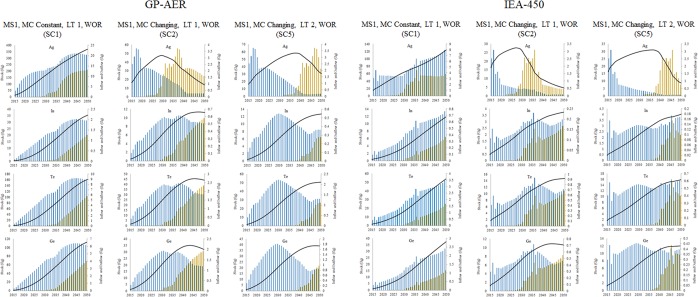
Inflows, outflows and stocks of selected PV solar metals in ***SC1, SC2, and SC5*** for GP-AER and IEA-450 GES.

Inflows, outflows and stocks of selected metals in PV technologies in ***SC1, SC2, and SC5***, for ***GP-AER*** and ***IEA-450 GES*** are shown in Fig. 7. Metals inflows, outflows, and stocks are showing different trends in different scenarios. In ***GP-AER, SC1***, stocks of metals are expected to increase overtime and inflows are expected to be larger than outflows untill 2050. In ***GP-AER, SC2***, stocks of Ag, Te, and Ge are expected to decrease overtime while stock of In is showing a sign of saturation around 2050 and inflows are expected to be smaller than outflows for all metals. In ***GP-AER, SC5***, stock of Ag is expected to decrease, stock of Ge is slightly decrease while stocks of In and Te are showing a sign of saturation around 2050, and inflows of Ag and Ge are expected to be smaller than the outflows, while inflows of In and Te larger than the outflow. Development of inflows, outflows, and stocks of metals indicates that when MC is changing and LT is short, discrded outflows, with prooper recycling system, could cover the demand for all metals towards 2050. This is not the case for all metals in the MC changing and long LT scenario. It is also clear that the decrese in the stock start earlier in the short LT. In ***IEA-450, SC1***, stocks of metals are expected to increase overtime and inflows are expected to be larger than outflows untill 2050. In ***IEA-450, SC2***, stocks of Ag and Ge are expected to decrease overtime while stocks of In and Te are slightly increasing towards 2050, and inflows are expected to be smaller than outflows for Ag and Ge and larger than outflows for In and Te. In ***IEA-450, SC5***, Ag stock is expected to decrease, Ge stock is showing sign of saturation, while In and Te stocks are expected to increase towards 2050, and Ag inflow is expected to be smaller than the outflow, while In, Te, and Ge inflows larger than the outflows. This indicates that in the longer term, the GES that high share of PV could be better in terms of the circular economy if the demand for metals is met initially.

**Figure 8:**
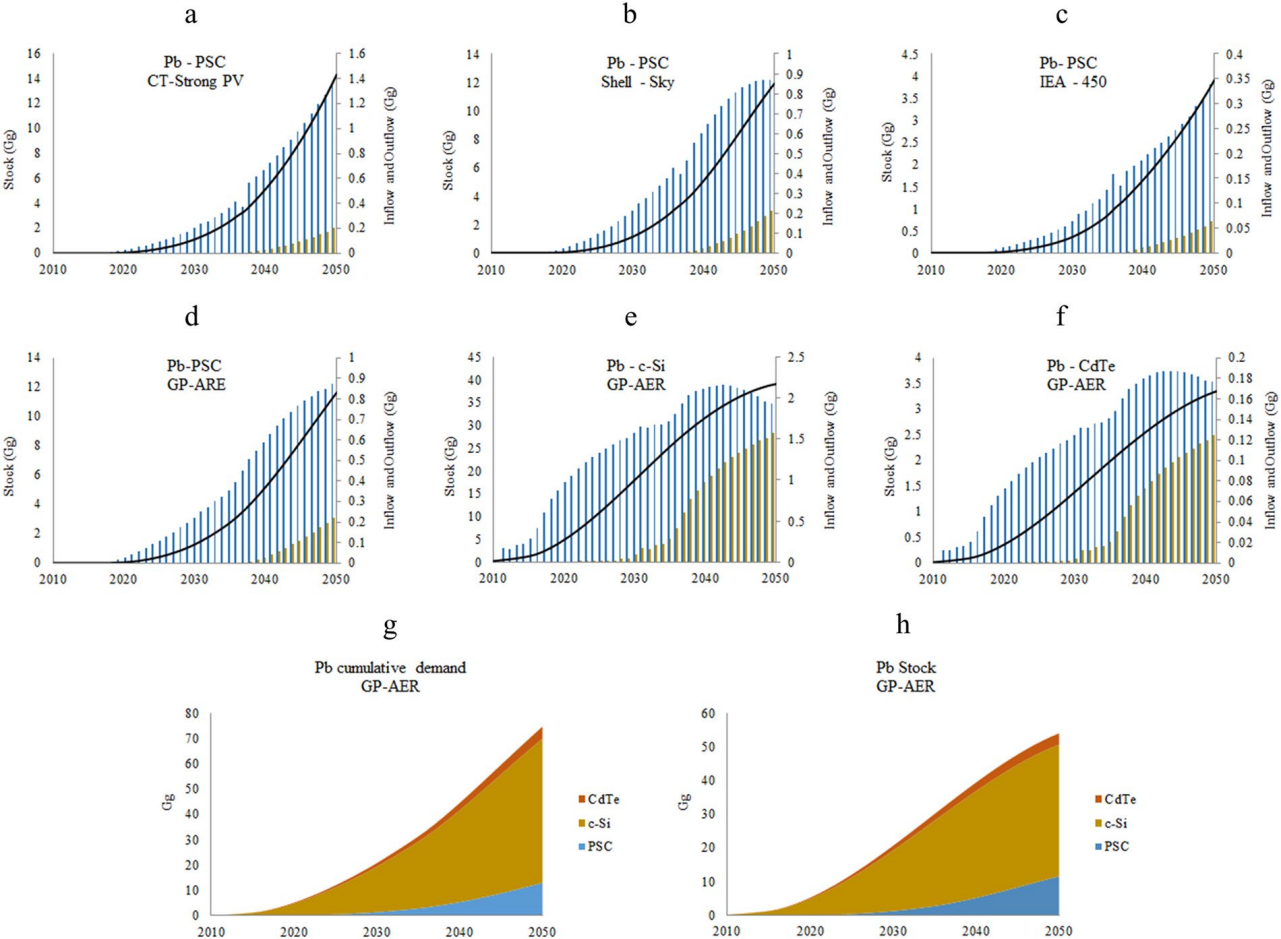
Inflows, outflows and stocks of lead in PSC in (**a**) CT-Strong PV, (**b**) Shell-Sky, and (**c**) IEA-450 and MS2, LT1, WOR scenarios; inflows, outflow and stocks in GP-AER in (**d**) PSC, (**e**) c-Si, and (**f**) CdTe; and (**g**) lead stocks and (**h**) cumulative demand in the three technologies in the GP-AER.

Inflows, outflows and stocks of lead in PSC in selcted GES and MS2, LT1, WOR scenarios are shown in Fig. 8a–c, flows, stocks, and cumulative demand of lead in PSC, c-Si, and CdTe technologies in ***GP-AER*** are shown in Fig. 8d–h in general the inflow and stock of lead by 2050 are expected to be between 160 Mg and 1370 Mg and between 1500 Mg and 14350 Mg in all GES. The stock of lead in PSC is expected to be about 30% of lead stock in c-Si and and 350% of lead stock in CdTe. This is attributed to lead content of these technologies and their market shares. Lead cumulative demand in PSC is expected to be 0.015% of its reserves and 0.3% of its current production, while lead cumulative demand in the three technologies is expected to be 0.1 of its reserves and 1.6 of its current production.

### PV technologies maximum contribution to future global energy system

Based on the above analysis, there are several factors determining possible market share of different PV technologies and ultimately PV solar market share in EG. These include technologies MC and LT, materials RP, resources availability, production capacity, and companion to host metals production ratios. In ***SC1 to SC6***, the determinant factor for CIGS is In resource availability, while for CdTe and a-Si, the determinant factor is Te and Ge production capacity. In ***SC7 and SC8***, the determinant factor for CIGS, has been shifted to In production capacity and for CdTe, the determinant factor has been shifted to Te resource availability.

**Figure 9:**
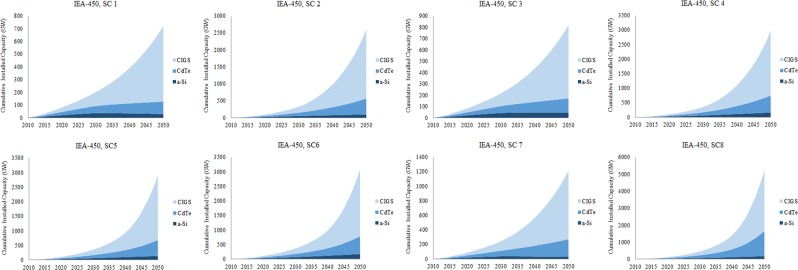
Installed capacities of CIGS, CdTe, and a-Si by 2050 under different materials scenarios.

**Figure 10:**
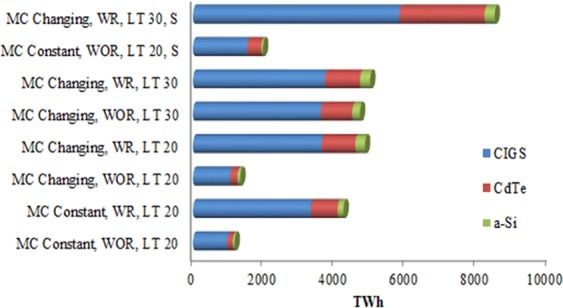
The maximum electricity generation by CIGS, CdTe, and a-Si by 2050 under different materials scenarios.

Installed capacities of CIGS, CdTe, and a-Si over time in IEA-450 scenario and different material scenarios based on the dynamic material flow-stock model and under different determinant factors have been estimated and shown in Fig. 9. The maximum EG by CIGS, CdTe, and a-Si by 2050 under different materials scenarios are shown in Fig. 10. The installed capacity of CIGS, CdTe, and a-Si could reach 23.4%, 84.6%, 26.6%, 96.6%, 93.8%, and 99.5% of PV installed capacity in IEA-450 scenario by 2050 in ***SC1*** to ***SC6*** respectively. Their market share could be up to 30%, 141.8, 34.5%, 163.6%, 159.6%, and 170% of PV market in IEA-450 scenario by 2050 in ***SC1*** to ***SC6*** respectively. To examine the impact of resources availability and production capacity on the maximum contribution of the TF technologies, ***SC7 and SC8*** assume different reserves for indium and production capacity for Te; the two metals constraining CIGS and CdTe technologies and applied to the original worst case scenario (***SC1***) and best case scenario (***SC6***). In ***SC 7***, if we added the contribution of a-Si technology in the original scenario (***SC1***) to CIGS and CdTe, TF technologies could reach 39.3% of PV installed capacity in IEA-450 scenario by 2050, and their market share could be up to 56.2% of PV market. In ***SC 8***, if we added the contribution of a-Si technology in the original scenario (***SC6***) to CIGS and CdTe, TF technologies could reach 169.4% of PV installed capacity in IEA-450 scenario by 2050, and their market share could be up to 322.4% of PV market. Overall the three TF technologies could reach between 23.4% and 170% of PV installed capacity, and their market share could be between 30% and 322% of the PV market by 2050 in IEA-450 scenario and all material scenarios. Maximum c-Si installed capacity by 2050 as determined by Ag resource availability could reach more than 100% of PV installed capacity without resource constraints in all material scenarios. The three TF technologies could provide between 23% and 39% of EG by PV solar in the IEA-450 scenario by 2050 if MC is constant and between 85% and 170% if MC is changing. The three technologies could provide between 3% and 5% of total EG in the IEA-450 scenario by 2050 if MC is constant and between 11% and 22% if MC is changing.

**Figure 11:**
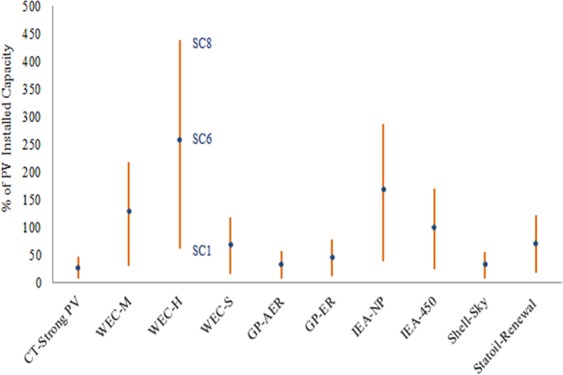
Installed capacity of CIGS, CdTe, and a-Si in ***SC1, SC6***, and ***SC8*** by 2050% of the total PV installed capacity in all GES.

In other GES (Fig. 11), based on the values of the maximum installed capacity of the three TF technologies in IEA-450 scenario and material scenarios ***SC1, SC6, and SC8***, these technologies could reach between 7% and 48% of the required PV installed capacity by 2050 in CT-Strong scenario; the highest PV energy scenario, and between 61% and 258% in WEC-H scenario; the lowest PV energy scenario.

The maximum electricity that could be provided by the three TF technologies by 2050 is between 1170 TWh and 4987 TWh (4.2 EJ and 18 EJ) in ***SC1*** and ***SC6*** respectively, or between 1970 TWh and 8490 TWh (7 EJ and 30 EJ) in ***SC7*** and ***SC8*** respectively (Fig. 10). The IPCC has estimated the electricity provided by solar to be between 12.2 EJ and 39 EJ by 2050 in the intermediate stabilization category where CO_2_ concentration between 440 and 600 ppm and in the most ambitious stabilizing scenario category where CO_2_ concentration remain below 440 ppm^78^. The three TF technologies could provide between 34% and 245% of solar energy in the intermediate stabilization category and between 11% and 77% of solar energy in the most ambitious stabilizing scenario category.

### Energy and water required for metals production and associated CO_2_ emissions

**Figure 12:**
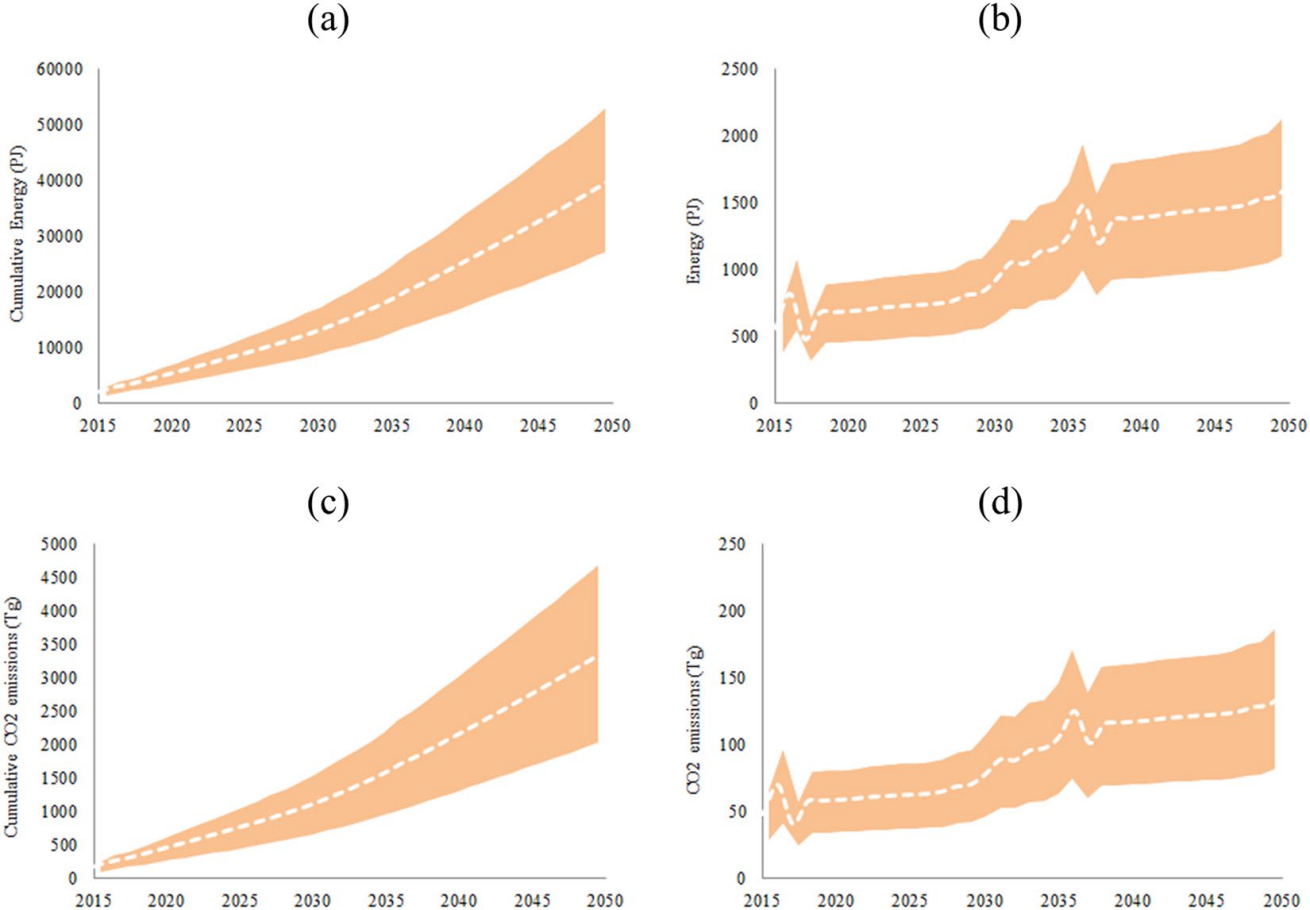
Cumulative energy required for all materials (**a**), annual energy required for all materials (**b**), cumulative CO_2_ emissions (**c**), and annual CO_2_ emissions (**d**), in IEA-450 scenario, ***SC1***, and the minimum, maximum, and average intensity of Al, steel, and concrete.

Based on materials demand and energy required for each material production, total cumulative amount of energy required for the production of all materials in IEA 450 scenario by 2050 is expected to reach between a minimum of 27120 PJ and a maximum of 52890 PJ in ***SC1*** (Fig. 12a) and between 26460 PJ and 52230 PJ in ***SC2*** in the minimum and maximum materials intensities. Total annual amount of energy required for all materials production in IEA-450 scenario by 2050 is expected to reach between a minimum of 1100 PJ and a maximum of 2130 PJ in ***SC1*** (Fig. 12b) and between 1065 PJ and 2094 PJ in ***SC2*** in the minimum and maximum materials intensities. The white dotted line in the Fig. 12 is the average amount of energy required for all materials production. The difference between the two scenarios is attributed to changing MC for minor metals in ***SC2***. These amounts are between 6.1% and 11.8% in ***SC1*** and between 5.9% and 11.6% in ***SC2*** of electricity generated by PV solar in IEA-450 scenario, and between 0.79% and 1.52% in ***SC1*** and between 0.76% and 1.5% in ***SC2*** of total electricity generated in IEA-450 scenario by 2050. Total cumulative CO_2_ emissions in the production of all materials in IEA 450 scenario by 2050 is expected to reach between a minimum of 2036 Tg and a maximum of 4685 Tg in ***SC1*** (Fig. 12c) and between 1997 Tg and 4646 Tg in ***SC2*** in the minimum and maximum materials intensities. Total annual CO_2_ emissions in the production of all materials in IEA-450 scenario by 2050 is expected to reach between a minimum of 82 Tg and a maximum of 187 Tg in ***SC1*** (Fig. 12d) and between 80 Tg and 185 Tg in ***SC2*** in the minimum and maximum materials intensities. These amounts are between 0.96% and 2.2% in ***SC1*** and between 0.94% and 2.16% in ***SC2*** of total CO_2_ emissions in IEA-450 scenario by 2050. Total amount of water required for the production of all materials in IEA 450 scenario by 2050 is expected to reach 0.597 bcm in ***SC1*** and 0.593 bcm in ***SC2*** by 2050 in the average materials intensities. Total amount of water required for the operation of PV solar in IEA 450 scenario is expected to reach 1.001 bcm in ***SC1*** and ***SC2***. The total water for PV solar materials and operational is expected to be 0.23% of the total water required for energy in the IEA-450 scenario.

**Figure 13:**
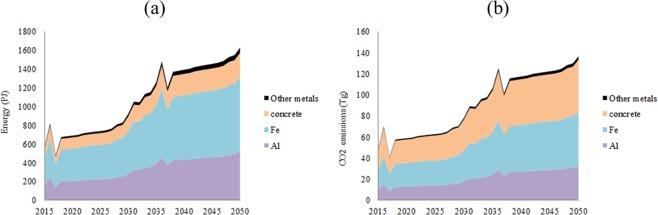
Annual energy required for all materials (**a**), and annual CO_2_ emissions (**b**), in IEA-450 scenario, ***SC1***, and the average intensity of Al, steel, and concrete.

Energy required for Al, steel, concrete, and all other metals in the average intensities of Al, steel, and concrete in ***SC1*** is shown in Fig. 13a and CO_2_ emissions in Fig. 13b. Energy required for Al production is expected to be between 35% and 31%, for steel production between 51% and 47.2%, for concrete between 8.8% and 19.1%, and for all other metals between 5.2% and 2.7% of total energy required for materials production in the minimum and maximum intensities of Al, steel, and concrete respectively in ***SC1***. Energy required for Al production is expected to be between 35.9% and 31.5%, for steel production between 53% and 48%, for concrete between 9% and 19.5%, and for all other metals between 2.1% and 1.0% of total energy required for materials production in the minimum and maximum intensities of Al, steel, and concrete respectively in ***SC2***. CO_2_ emission for Al production is expected to be between 29.1% and 22.1%, for steel production between 44.7% and 34.9%, for concrete between 22.2% and 41.3%, and for all other metals between 4.0% and 1.7% of total CO_2_ emissions in the minimum and maximum intensities of Al, steel, and concrete respectively in ***SC1***. CO_2_ emission for Al production is expected to be between 29.9% and 22.3%, for steel production between 45.8% and 35.3%, for concrete between 22.8% and 41.7%, and for all other metals between 1.5% and 0.7% of total CO_2_ emissions in the minimum and maximum intensities of Al, steel, and concrete respectively in ***SC2***.

## Discussion

The analysis presented in this paper investigates 10 GES. Although most of these scenarios are aimed at achieving CO_2_ emissions reduction to keep the temperature increase below the 2 degree, energy supply and demand structures are different in these scenarios, and consequently CO_2_ emissions. Two of the investigated scenarios; CT-Strong PV and Shell-Sky, have the highest market share of PV solar but also have the highest CO_2_ emissions, due to electricity mix in first and to electricity total demand and fossil fuel share in the second.

Several estimates for the potential contribution of PV technologies have been reported^16,39–42^. Cumulative installed capacity for both CIGS and CdTe is estimated between 240 GW^40^ and 11,000 GW^41^. Cumulative installed capacity of CdTe is estimated in the range of 600 to 2700 GW^42^ and 200 to 4000 GW^39^. Installed capacity of c-Si, CIGS and CdTe is estimated as 1850 GW by 2030^16^. The difference in these estimates is attributed to the used approach, assumptions, and evaluation criteria. The results obtained in this study for the maximum installed capacity of the three TF technologies are between these results. The analysis shows however, that the limiting factor is not the same for all technologies and could change for specific technology if new information appears.

The analysis also shows that it is difficult to realize most of the energy scenarios with commercially available PV technologies and their current conditions. This is in contrast to the argument used in literature^11,12^. However, several measures can be taken to reduce the risk associated with material availability and production capacity including reducing the amount of materials used in PV solar technologies and increasing their recycling rate, extending the LT of these technologies, and increasing the effort in finding new technologies, increasing their efficiencies, and reducing their use of critical materials. These aspects are illustrated by the different scenarios. It is clear that the most significant factor is technologies material content followed by the availability of metals recycling system and technologies LT. It is also clear that careful policies for metals and technologies have to be made. A complete phase out of CdTe has been assumed in a scenario used to evaluate material requirements for the German energy system^17^. Although the analysis described here shows that the contribution of CdTe to PV solar market is limited, the phase out should be carefully addressed due to the fact that Cd availability is supply driven^37^. The need for indium and Ge in the other two TF technologies will lead to an increase in Zn production and consequently Cd production as a companion metal. CdTe technology could therefore be seen as a better option for Cd use instead of its disposal.

One of the options to increase the potential contribution of PV solar to future EG is diversifying technologies within the PV solar market. This option is illustrated in the analysis by the second market of PV technologies (MS2). Although this market has no restrictions in terms of resources, especially when technological development reduces PV technologies MC, there are other concerns associated with this market due to the market share of PSCs. PSCs are emerging PV technologies with certified efficiency of more than 23%^79^. The outlook of these cells is determined by three main factors; energy conversion efficiency, cost and use of specific material including gold and lead, and stability^80^. The use of PSCs has several advantages in terms of cost and efficiency. The highest efficiency perovskites are Pb-based^81^. Although the use of lead, which is in the range of 7 to 10 kg/MW^80,82^, has some advantages, it could be of concern. The advantage is related to the possibility of using discarded lead from end of life lead-acid batteries, which are expected to be replaced by lithium based and other batteries^83^, with low energy and other environmental impacts compared to lead mining. In addition, it provides alternative management option for the disposal of lead-acid batteries. Major concern is the possibility of lead leaching to the environment and ground water^82^. Long-term device and material stability and up scaling are currently the main limitations and concerns^79,84^. Adding more inorganic elements is increasing perovskite complexity and an addition of rubidium (Rb) is proposed to create perovskite materials with excellent material properties^81^. Other metals are proposed for lead-free organic inorganic halid perovskite solar cells, including tin, germanium, and bismuth. Efficiencies of technologies based on these materials however are very low and tin-based technology has larger environmental impacts^82^. Meanwhile, the use of metals to improve long-term device and material stability including rubidium (Rb) could be of concern. Although advances in material science may allow these technologies to be part of the PV market, most of proposed materials have resource limitation. Ge is produced in low quantities and as a companion of zinc (see the analysis on a-Si PV solar technology). Bi is mainly produced with lead and in light of the shift in the batteries market, it is expected that recycled material to replace lead mining. Rb is also produced in small quantities from primary resources only, as a by-product of other metals (Cesium, lithium, and strontium), and mainly in two countries (Namibia, and Zimbabwe)^57^.

The analysis carried here could be enhanced by including either dynamic material flow analysis bottom-up or top-down approaches for the metals used in the PV solar technologies. This would allow for estimating the future demand for these metals not only for PV solar technologies but also for other applications. This would be important for metals that are widely used in other applications such as In, while less important for other metals including Te and Ge since their use in other applications is relatively low^16,37^. In addition, two of the most significant factors are resources availability and production capacity. While production capacity to a large extent has reliable data, estimates for resource availability are less reliable. Weather uncertainties in these estimates are due to politics, economics or corporate confidentiality^60^, accurate and transparent estimates of resources is necessary.

PV solar materials production uses a considerable amount of the energy produced by these technologies attributed mainly to Al, steel, and concrete, although other metals will also use some of this energy. Similar to energy, PV solar materials production is associated with a considerable amount of CO_2_ emissions. The main factors determining the energy demand and CO_2_ emissions are materials intensity of technologies, and energy and emissions intensities. The analysis includes a sensitivity analysis of Al, steel, and concrete intensities and a scenario for energy and CO_2_ intensities of the three materials. For more accurate results of the estimates of energy and emissions however, it is important to include a dynamic analysis of energy and emissions intensities based on material quality, energy efficiency, energy mix, and other factors^26,85^.

Finally the analysis carried here is applied for PV solar technologies, their contribution to global energy system, and energy, water, and CO_2_ emissions associated with their materials production, however, the methodology can be applied to other energy technologies and other sectors.

## Conclusions

The analysis in this paper is carried out based on a dynamic material flow-stock model and a total of 100 scenarios, which combine GES and material scenarios. These scenarios have been designed to investigate the impacts of different factors (MS, MC, RP, and LT) on the demand for metals, and (resources availability and production capacity) on their supply, and ultimately the possible realization of the GES. The evaluation is carried out based on a comparison between metals cumulative demand (CD) and their global reserves, average annual demand (AAD) and their global production, and required production of host metals and their historical production growth rate. Based on the analysis, the maximum contribution of each technology, energy and water required for materials production and associated CO2 emissions are estimated. Several conclusions can be drawn from the analysis

- Each PV solar technology has limiting metal; Ag for c-Si, In for CIGS, Te for CdTe, and Ge for a-Si.
- Although the realization of most of the GES is difficult under current PV technologies market shares and conditions, these technologies could make significant contribution to EG in the future.
- The main determinant factor of future demand for PV solar technologies metals is technologies MC followed by materials RP and technologies LT.
- The determinant factors for PV sub-technologies on the supply side of materials are not always the same (e.g. production capacity in the case of CdTe and a-Si, and resources availability in CIGS), and could change under certain conditions of the technology (e.g. the determinant factor for CIGS has been shifted from resource availability to production capacity).
- The development of metals inflow, outflow and stocks indicates that, when technologies MC is decreasing and the LT is short, metals outflows are expected to exceed their inflows. With proper recycling system, metals demand could be met by secondary sources towards 2050.
- Although global energy scenarios with high market share of PV solar requires more metals in the next 30 years, these scenarios on the long term could be better if metals demand from primary sources is secured combined with increasing resource efficiency and recycling.
- Although Perovskite solar cells has no resource limitation, alternative materials for lead free technology and the use of critical materials to improve their stability should be carefully addressed.
- PV solar technologies materials production is expected to use a considerable amount of the energy produced by these technologies and to be associated with a considerable amount of the expected CO_2_ emissions in the scenario.

## Supplementary information

Supplementary Information

## Data Availability

The data used in the analysis is either available online in the reports published on the scenarios developers and United States Geological Survey (USGS) websites or available from the corresponding author upon request.
