# Supplementary material for: Materials, energy, water, and emissions nexus impacts on the future contribution of PV solar technologies to global energy scenarios
Source: Sci Rep. 2019 Dec 17;9:19238. doi: 10.1038/s41598-019-55853-w (PMC6917800; doi:10.1038/s41598-019-55853-w)
Supplement: Supplementary file 1 — Supplementary Information [file 41598_2019_55853_MOESM1_ESM.docx]

Materials, energy, water, and emissions nexus impacts on the future contribution of PV solar technologies to global energy scenarios

Ayman Elshkaki^a*^

^a^ Institute of Geographic Sciences and Natural Resources Research, Chinese Academy of Sciences, 11A Datun Road, Chaoyang District, Beijing 100101, PR China

* Corresponding author. Tel.: +86-13261017251; Fax: +86-1064889005

E-mail address: [ayman@igsnrr.ac.cn;](mailto:ayman@igsnrr.ac.cn;) elshkaki@gmail.com

ORCID ID: 0000-0003-4602-0974

**Supplementary Information**


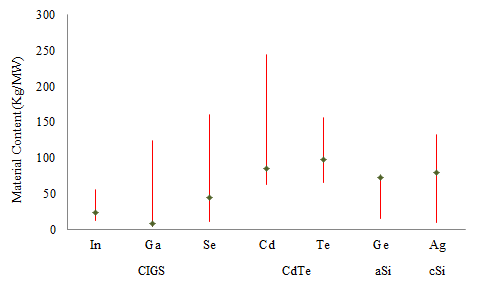


Fig S1: The minimum and maximum technologies material content reported in literature and assumed values for technologies material content constant scenario in this study

Table S1: Material requirements for different PV solar technologies (kg/MW) reported in literature

|  | [1] | [2] | [3]^1^ | [4] | [5] | [6]^2^ | [7] | Range |
| --- | --- | --- | --- | --- | --- | --- | --- | --- |
| c-Si |  |  |  |  |  |  |  |  |
| Ag | 10 | 80 | 84 | 59-68 |  | 133 | 36 | 10 - 133 |
| Cu | 884 |  |  | 825-943 |  | 4177.5 |  | 825 - 4177.5 |
| CdTe |  |  |  |  |  |  |  |  |
| Te | 70 | 97.5 | 101 | 243 | 99.7 | 65.4 | 156 | 65.4 - 156 |
| Cd | 62 | 85 | 89 | 244 | 116.7 | 65.2 | 138 | 62 - 244 |
| Cu | 5181 |  |  | 5181 |  | 42.8 |  | 42.8 - 5181 |
| CIGS |  |  |  |  |  |  |  |  |
| In | 13 | 23 | 29 | 55 | 55 | 23.2 | 28 | 13 - 55 |
| Ga | 4 | 7.5 | 8 | 124 | 7.2 | 4.9 | 9 | 4 - 124 |
| Se | 41 | 45 | 67 | 11 | 39.3 | 38.1 | 161 | 11 - 161 |
| Cu | 450 |  |  | 450 |  | 19 |  |  |
| a-Si |  |  |  |  |  |  |  |  |
| In | 5.32 |  |  | 13 | 4 |  |  | 4 - 13 |
| Ge |  |  | 73 |  |  | 14.8 |  | 14.8 - 73 |
| Cu | 1005 |  |  | 1005 |  |  |  | 1005 |

1 The values are for the year 2010

2 The values are the average compiled by the authors based on different studies

Table S2: Assumption on PV solar technologies market share in different studies

| Study | PV solar technologies market share |
| --- | --- |
| [2] | Three scenarios have been assumed (reference scenario, high scenario and low scenario) for the market share of CIGS and CdTe solar panels with a maximum share of 20%, 30%, and 50% within the PV market by 2050. |
| [3] | Two technologies market share have been assumed; the first assumes a market share that remains at current levels with c-Si share of 85% and the three thin-film technologies have a market share of 5% each, the second assumes one thin-film technology to increase its share from 5% to 50%, while the other two thin-film technologies remains at 5% share each and the market share of the c-Si technology to decrease from 85% in to 40%. |
| [5] | Two scenarios for PV solar technologies have been used. The first assumes that the main technology will be c-Si with less than 5% market share of thin film technologies, and the second assumes a 42% market share of thin film technologies in 2050 and constitutes only CIGS and a-Si technologies. |
| [6] | The market of PV solar is assumed to be mainly dominated by crystalline technologies with a share of 85%, and each of the thin film technologies (CIGS, CdTe and a-Si) has a market share of 5%. |
| [7] | Two share patterns have been used; the first assumes 100% c-Si, and the other assumes 50/50% of CIGS/CdTe over the time horizon. |

|  |  |
| --- | --- |

Fig S2: Historical ratios of companion metals to host metals production

Fig. S3: Historical world production of Cu, Zn and their companion metals as a function of time

References

1. Manberger, A., Stenqvist, B., 2018. Global metal flows in the renewable energy transition: Exploring the effects of substitutes, technological mix and development. Energy Policy, 119, 226-241
2. Watari, T., McLellan, B. C., Ogata, S., Tezuka, T., 2018. Analysis of Potential for Critical Metal Resource Constraints in the International Energy Agency’s Long-Term Low-Carbon Energy Scenarios. Minerals, 8, 156; doi:10.3390/min8040156
3. Nassar, N., Wilburn, D. R., Goonan, T. G., 2016. Byproduct metal requirements for U.S. wind and solar photovoltaic electricity generation up to the year 2040 under various Clean Power Plan scenarios. Applied Energy, 183, 1209-1226.
4. Florian Fizaine, Victor Court, Renewable electricity producing technologies and metal depletion: A sensitivity analysis using the EROI. Ecological Economics, 2010, 106-118.
5. Viebahn, P., Soukup, O., Samadi, S., Teubler, J., Wiesen, K., Ritthoff, M., 2015. Assessing the need for critical minerals to shift the German energy system towards a high proportion of renewables. Renewable and sustainable Energy Reviews, 49, 655-671.
6. Valero, A., Valero, A., Calvo, G., Ortego, A., 2018. Material bottlenecks in the future development of green technologies. Renewable and Sustainable Energy Reviews, 93, 178-200.
7. Tokimatsu, K., Wachtmeister, H., McLellan, B., Davidsson, S., Murakami, S., Höök, M., Yasuoka, R., Nishio, M., 2017. Energy modeling approach to the global energy-mineral nexus: A first look at metal requirements and the 2 _C target. Applied Energy, 207, 494-509
